# Using the Inflammacheck Device to Measure the Level of Exhaled Breath Condensate Hydrogen Peroxide in Patients With Asthma and Chronic Obstructive Pulmonary Disease (The EXHALE Pilot Study): Protocol for a Cross-Sectional Feasibility Study

**DOI:** 10.2196/resprot.8768

**Published:** 2018-01-30

**Authors:** Daniel M Neville, Carole Fogg, Thomas P Brown, Thomas L Jones, Eleanor Lanning, Paul Bassett, Anoop J Chauhan

**Affiliations:** ^1^ Department of Respiratory Research & Innovation Portsmouth Hospitals NHS Trust Portsmouth United Kingdom; ^2^ Stats Consultancy Ltd Amersham United Kingdom

**Keywords:** reexhalation, biomarkers, medical device, asthma, COPD

## Abstract

**Background:**

Asthma and Chronic Obstructive Pulmonary Disease (COPD) are common conditions that affect over 5 million people in the United Kingdom. These groups of patients suffer significantly from breathlessness and recurrent exacerbations that can be difficult to diagnose and go untreated. A common feature of COPD and asthma is airway inflammation that increases before and during exacerbations. Current methods of assessing airway inflammation can be invasive, difficult to perform, and are often inaccurate. In contrast, measurement of exhaled breath condensate (EBC) hydrogen peroxide (H_2_O_2_) is performed during normal tidal breathing and is known to reflect the level of global inflammation in the airways. There is a need for novel tools to diagnose asthma and COPD earlier and to detect increased airway inflammation that precedes an exacerbation.

**Objective:**

The aim of this study was to explore the use of a new handheld device (called Inflammacheck) in measuring H_2_O_2_ levels in EBC. We will study whether it can measure EBC H_2_O_2_ levels consistently and whether it can be used to differentiate asthma and COPD from healthy controls.

**Methods:**

We will perform a cross-sectional, feasibility, pilot study of EBC H_2_O_2_ levels, as measured by Inflammacheck, and other markers of disease severity and symptom control in patients with asthma and COPD and volunteers with no history of lung disease. Participants will be asked to provide an exhaled breath sample for measurement of their EBC H_2_O_2_ using Inflammacheck. The result will be correlated with disease stage, spirometry, fractional exhaled nitric oxide (FeNO), and symptom control scores.

**Results:**

This study’s recruitment is ongoing; it is anticipated that the results will be available in 2018.

**Conclusions:**

The EXhaled Hydrogen peroxide As a marker of Lung diseasE (EXHALE) pilot study will provide an evaluation of a new method of measuring EBC H_2_O_2_. It will assess the device’s consistency and ability to distinguish airway inflammation in asthma and COPD compared with healthy controls.

## Introduction

### Burden of Chronic Obstructive Pulmonary Disease and Asthma

Chronic Obstructive Pulmonary Disease (COPD) is a common and treatable condition that is characterized by predominately irreversible and progressive airflow limitation. COPD is a leading cause of morbidity and mortality worldwide, and its prevalence is predicted to increase substantially in the coming decade [1]. In the United Kingdom alone, COPD affects an estimated 3 million people, with two-thirds remaining undiagnosed [2,3]. Delays in reaching an accurate diagnosis impact on people’s quality of life (QoL) and health care resource utilization. COPD is associated with a significant economic and social burden. In 2011, there were over a million in-patient bed days caused by acute exacerbations of COPD, with a severe exacerbation requiring admission to hospital costing up to £1600, therefore having a major impact on health care expenditure [4]. COPD also has a personal burden, causing 24 million lost working days annually, costing the UK economy £3.8 billion [5,6].

Asthma is estimated to affect over 3.5 million people in the United Kingdom, with 250,000 experiencing severe disease with frequent exacerbations [7]. Despite increasing numbers of treatment options, there were still over 900 deaths because of asthma in 2014 [8]. The economic burden of treating asthma is huge, costing in excess of £1 billion per year [4,7]. Like COPD, asthma also has a dramatic personal cost with 1 in 5 asthmatics in the United Kingdom reporting serious concerns that their next asthma attack will kill them [9]. Delays in correctly diagnosing and phenotyping asthma can lead to poor disease control, high emergency health care use, and inappropriate treatment [10,11]. There is a need for additional, widely accessible tools to aid in the accurate diagnosis and phenotyping of both asthma and COPD.

### Rationale for Measuring Airway Inflammation

A common feature of COPD and asthma is chronic airway inflammation, which worsens during exacerbations. These are frequently triggered by infections and inhaled irritants resulting in epithelial injury, neutrophil and eosinophil activation, and release of inflammatory cytokines [10,12]. This all contributes to the bronchoconstriction, smooth muscle hypertrophy, and airflow obstruction that are features of asthma [13]. Where there is prolonged exposure to toxic irritants in COPD, there is ongoing airway inflammation that can continue even after smoking or irritant cessation and begin before the development of clinical symptoms [1,14].

Airway inflammation in asthma and COPD leads to an imbalance between the production of reactive oxygen species (ROS) and the ability of the body to counteract their harmful effects through neutralization by antioxidants; this is termed as oxidative stress [15-17]. Increased expression of ROS by activated inflammatory cells, including neutrophils and eosinophils, can lead to further generation of inflammatory mediators, causing damage to epithelial cells and increased bronchial hyperreactivity [14]. ROS are metabolized in cells to produce highly reactive oxidants such as hydrogen peroxide (H_2_O_2_), which is fat soluble and can move across cell membranes. As H_2_O_2_ is volatile and readily equilibrates with air, its presence can be detected in exhaled breath condensate (EBC). Therefore, measurement of EBC H_2_O_2_ gives a quantitative measure of oxidative stress and airway inflammation [18-21].

### Current Measures of Airway Inflammation

The current *gold standard* tool for assessing airway inflammation and oxidative stress is fiberoptic bronchoscopy with bronchial wall biopsy and bronchial fluid lavage [22,23]. This is an invasive procedure that is not suitable for routine clinical practice or regular repeat sampling. This is because it carries a small but important risk, with complications occurring in up to 4.3% of procedures and a reported procedure-related mortality of up to 0.1% [24]. In patients with underlying asthma and COPD, the risk is even greater, with up to 10% of patients with asthma developing respiratory symptoms post bronchoscopy [24]. Bronchoscopy is also an expensive tool, with British Thoracic Society guidelines recommending a minimum of two qualified nurses present throughout bronchoscopy procedures and one qualified nurse to recover a patient post bronchoscopy [24]. Furthermore, sample analysis requires a series of laboratory measurements, and results can take over 24 hours to become available, causing delays in clinical decision making.

Induced sputum analysis is a semi-invasive means of assessing airway inflammation [25]. However it can be unpleasant, technically demanding, and time consuming, and as a result, is not always possible in patients with more severe airflow obstruction and poor lung function. It is not always well tolerated by patients and is not suitable for repeat sampling [26]. Noninvasive means of assessing airway inflammation presently measure fractional exhaled nitric oxide (FeNO—a specific measure of eosinophilic airway inflammation) [27]. This requires controlled exhalation for at least 6 seconds, making the test unsuitable for patients with significantly impaired lung function and especially those who are tachypnoeic during an exacerbation. Furthermore, FeNO is lowered in current smokers, limiting its diagnostic use, and it also does not measure neutrophilic airway inflammation, a recognized component of COPD and steroid insensitive asthma. It has been reported that up to 50% of patients with severe asthma do not have eosinophilic-driven disease (noneosinophilic) and, as a consequence, FeNO cannot monitor management in this group [28].

### Exhaled Breath Condensate Hydrogen Peroxide

EBC contains aerosolized particles from the airway epithelial lining fluid [29], including volatile water-soluble compounds such as H_2_O_2_. Measurement of EBC H_2_O_2_ therefore gives a direct, quantitative measure of airway inflammation [21,30,31]. In contrast to current measures of airway inflammation, collection of EBC H_2_O_2_ is performed during tidal breathing, making it noninvasive and easy to perform. It can be repeated quickly and is well tolerated even in patients with severe airways disease. It is widely appreciated that EBC H_2_O_2_ measurement has the potential to improve clinical practice by safely providing vital information on aspects of disease that are currently inaccessible [32].

To date, measurement of H_2_O_2_ in the EBC has required complex, multi-step processing of the collected breath samples to produce a result and as a consequence has largely been used as a research tool [33]. The evidence from these past collection techniques show that EBC H_2_O_2_ levels are significantly higher in COPD patients compared with healthy controls [34]. It has also been demonstrated that the levels rise further during COPD exacerbations, and there is some evidence that EBC H_2_O_2_ levels correlate with COPD disease severity [21]. Within asthma, EBC H_2_O_2_ concentrations were significantly higher in asthmatics who were nonsmokers compared with healthy subjects [30,35]. The level of EBC H_2_O_2_ also correlated with asthma severity and phenotype, being significantly higher in moderate asthmatics compared with those with mild asthma and in asthmatics with neutrophil predominant airway inflammation [36,37]. Furthermore, higher values of EBC H_2_O_2_ were observed in uncontrolled asthma (defined as increased use of short-acting beta-agonist and continued daily symptoms) compared with healthy subjects and controlled asthmatics [31].

Exhalation Technology Ltd. has developed a battery operated, handheld device for point of care measurement of H_2_O_2_ level in exhaled breath—the Inflammacheck device. This test involves simple, relaxed tidal breathing for up to 1 min (20 breaths) into a mouthpiece. The device collects at least 60 µL of EBC in a collection cartridge. A total of 30 µL of the collected EBC is then pipetted by the clinician onto a separate sensor cartridge that measures the level of H_2_O_2_. If this device can be used in a routine clinical setting, it may give clinicians an immediate insight into the inflammatory state of the airways. It also has the potential to identify inflammatory cell specific inflammation (neutrophilic) that would guide treatment decision making. This could aid earlier diagnosis and personalized management plans, ultimately improving patient care. This simple, noninvasive technique may also make repeat sampling and longitudinal monitoring of global airway inflammation a realistic possibility.

We aim to assess whether Inflammacheck can differentiate asthma and COPD from healthy airways and whether its measurement of EBC H_2_O_2_ correlates with other noninvasive methods of assessing airway inflammation and disease severity. Information about the reliability and repeatability of the Inflammacheck sensor also needs to be collected. We will assess Inflammacheck in patients with asthma, COPD, and healthy individuals who do not have respiratory disease.

### Aims and Objectives

#### Coprimary Objectives

The coprimary objectives were as follows:

- To determine whether Inflammacheck can differentiate asthma and COPD from healthy controls.
- Whether Inflammcheck can detect EBC H_2_O_2_ levels consistently and in a repeatable manner.

#### Secondary Objectives

The secondary objectives were as follows:

- To describe the relationship between EBC H_2_O_2_ and the following:
  - Disease severity (Global Initiative for Asthma [GINA] stage for asthma [38], Global Initiative for Obstructive Lung Disease [GOLD] stage for COPD [1])
  - Disease control (Asthma Control Questionnaire, ACQ score [asthma] and COPD Assessment Test, CAT score [COPD])
  - QoL (Asthma Quality of Life Questionnaire, AQLQ score [asthma])
  - Spirometry (forced expiratory volume in 1 second [FEV_1_], forced vital capacity [FVC], and ratio), with reversibility where available
  - FeNO (ppb)
  - Atopic status (asthmatics only)
- To determine the reliability and consistency of the Inflammacheck sensor in measuring EBC H_2_O_2_ levels with laboratory reference ranges.
- To determine how frequently patients are unable to perform spirometry, FeNO, and Inflammacheck, or require further attempts.
- To describe any adverse events during the test procedures.
- To explore participants’ and health care professionals’ (HCPs’) experience of Inflammacheck, including *ease of use* and acceptability of the device.

#### Exploratory Objectives

To determine whether there is a relationship between EBC H_2_O_2_, as measured by Inflammacheck and FeNO, atopic status (skin prick testing [SPT] result in asthmatics only), exercise, diet, medications, and effects of inhaled therapy. EBC H_2_O_2_ levels will also be compared with peripheral white cell counts, serum biochemistry, and markers of inflammation where available.

## Methods

### Overview

This is a single visit, cross-sectional, feasibility study of EBC H_2_O_2_ levels, as measured by Inflammacheck, and markers of disease severity and symptom control in patients with asthma, COPD, and volunteers with no known lung disease.

### Outcome Measures

Respiratory outcomes were as follows:

- EBC H_2_O_2_ as measured by the Inflammacheck sensor
- EBC H_2_O_2_ as measured by a reference and background sensor
- FeNO
- FEV_1_, FVC, and ratio.
- GINA stage in asthma patients, GOLD stage in COPD patients.
- ACQ and CAT scores
- AQLQ score

Process outcomes were as follows:

- Number of attempts at each procedure
- Whether patient successfully completed procedure

Safety outcome was as follows:

- Any adverse events reported during any of the study procedures

Experience outcomes were as follows:

- Participant’s perception of device
- HCP’s perception of device

Environmental and biological outcomes included the following:

- Atopic status (SPT result in asthmatics), diet, exercise, and medication use.

### Study Participants

Three populations of older adolescent and adult patients (all aged ≥16 years) will be invited to participate:

1. Asthma patients (n=30)
2. COPD patients (n=30)
3. Comparator group (n=30)—Volunteers with no previous history of lung disease (our healthy volunteers). These healthy volunteers will be recruited from the hospital staff. This will be facilitated by the hospital and research communications teams. HCPs, who have assisted patients performing Inflammacheck, alongside the standard respiratory tests of spirometry and FeNO, will be invited to participate in an experience outcome questionnaire at the end of the study.

### Inclusion and Exclusion Criteria

Inclusion criteria for the participants are provided in Textbox 1.

HCPs inclusion criteria were as follows:

- Assisted a minimum of 5 patients in performing the collection of EBC H_2_O_2_ levels using the Inflammacheck device during the study.
- Willing and able to give informed consent for participation in the study.

Exclusion criteria for the participants are provided in Textbox 2.

**Inclusion criteria for study participants.**

- Male or Female, aged ≥16 years.
- Any of the following conditions:
  1. A confirmed, clinician-made diagnosis of asthma with symptoms for ≥3 months supported by evidence of any of the following:
    - Airflow variability, with a variability in forced expiratory volume in 1 second (FEV_1_) or peak expiratory flow of >20% across clinic visits, with concomitant evidence of airflow obstruction (FEV_1_/FVC ratio <70% on spirometry recorded at any time);
    - Airway reversibility with an improvement in FEV_1_ by ≥12% or 200 mL after inhalation of 400 μg of salbutamol (or equivalent bronchodilator) via a metered dose inhaler and spacer or nebulizer, recorded at any time;
    - Airway hyper-responsiveness demonstrated by Methacholine challenge testing with a provocative concentration of Methacholine required to cause a 20% reduction in FEV_1_ (PC20) of ≤ 16 mg/mL or equivalent test, recorded at any time.
  2. *OR* a confirmed, clinician-made diagnosis of Chronic Obstructive Pulmonary Disease (COPD) for ≥3 months supported by spirometric evidence of fixed airflow limitation (postbronchodilator FEV_1_ / FVC ratio <0.7) recorded at any time.
  3. *OR* no known history of lung disease (defined as no current clinical diagnosis of, or be receiving treatment for, a lung disease).
- Willing and able to give informed consent for participation in the study.

**Exclusion criteria for study participants.**

The participant may not enter the study if any of the following apply:

- Existing comorbidities that may prevent them from performing spirometry, fractional exhaled nitric oxide (FeNO), or other study measurements (at the discretion of the clinical investigator).
- Known other lung, chest wall, neuromuscular, or cardiac disease or abnormality (including end-stage disease or cancer) that would confound symptom scores and spirometry.
- Has received treatment for an exacerbation of their respiratory disease within the last 2 weeks.
- In the opinion of the clinical investigator, participant could be put at risk of harm by having to perform any of the study procedures.
- Unable to comprehend the study and provide informed consent, for example, insufficient command of English in the absence of someone to adequately interpret.

### Sampling and Sample Size

The sample size was based on our primary objective of comparing EBC H_2_O_2_ values between each pair of the three main study groups (asthma patients, COPD patients, and controls). This is a feasibility pilot study of the use of a new device. For future, larger trials to be conducted using the device, information is needed to confirm that the Inflammacheck sensor is accurate and can consistently measure H_2_O_2_ in EBC within patients. There are no previous trials using this version of the device that can guide a sample size calculation. As a result, the sample size is based on the research teams’ experience of delivering previous trials on novel diagnostic tests.

### Study Procedures

#### Recruitment

##### Outpatients

Patients attending respiratory outpatient clinics at Queen Alexandra Hospital in Portsmouth will be will be preidentified from upcoming clinic lists and sent a participant information sheet (PIS) and an invitation to participate with their clinic letter. Patients will have adequate time to read this information, and there will be a contact telephone number and email address on the invitation letter so that potential participants are able to discuss the study with a member of the research team before their clinic visit. Patients who are already enrolled in other research trials will be invited and allowed to participate in the study if they so wish. For the recruitment of mild disease, patients will be identified from specialist respiratory clinics held in the community. They will be given a PIS with an invitation to participate in the study.

##### Healthy Volunteers

Posters advertising the study will be placed in staff rest rooms, and an email will be sent to all staff (through the hospital research communications team) to advertise the study. Potential volunteers will be asked to call or email the study team to so that further information (PIS) can be sent via an email and an appointment arranged to discuss the study further.

HCPs

HCPs and members of the research team within the respiratory department, who perform all respiratory tests on the participants, will be asked to participate within the study to answer a questionnaire on their perceptions of the Inflammacheck device. Only HCPs who have assisted a minimum of 5 patients in performing the collection of EBC H_2_O_2_ levels using Inflammacheck will be asked to participate.

### Screening and Enrollment

#### Outpatients

Those expressing an interest in the study will have eligibility assessed and the opportunity to ask any further questions on their clinic visit. Informed consent will then be taken.

#### Healthy Volunteers

An appointment will be made with the research team at a convenient time to confirm eligibility criteria and take informed consent.

#### HCPs

At the end of the study, the research team will approach all HCPs involved in assisting patients in performing the collection of EBC H_2_O_2_ levels using the Inflammacheck device. If HCPs who have assisted at least 5 participants in performing the study breathing tests express an interest in participating, informed consent will be taken.

### Study Assessments

**Figure 1:**
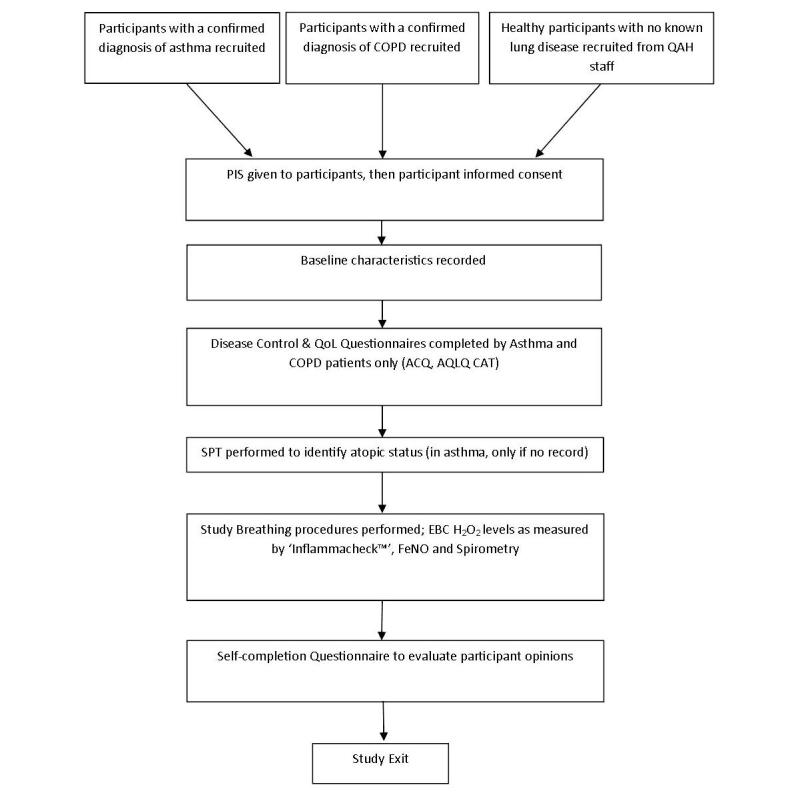
Summary of study procedures and the participant flow. COPD: Chronic Obstructive Pulmonary Disease; QAH: Queen Alexandra Hospital; PIS: participant information sheet; QoL: quality of life; ACQ: asthma control questionnaire; AQLQ: asthma quality of life questionnaire; CAT: COPD assessment test; SPT: skin prick test; EBC H_2_O_2_: exhaled breath condensate hydrogen peroxide; FeNO: fractional exhaled nitric oxide.

A summary of the study assessments and participant flow is displayed in Figure 1.

#### Participant Characteristics

Before other study procedures, characteristics of participants that are known to have a potential influence on respiratory test results will be documented. These will include the following:

- Demographics: age, gender, and ethnicity
- Anthropometry: Height and weight (to calculate body mass index)
- Disease severity: GINA stage (asthma), GOLD stage (COPD)
- Medications participants are taking
- Factors that may affect EBC H_2_O_2_ levels including smoking status (current or former or never), time of last cigarette (for current smokers only), number of pack years of smoking (current and former smokers only), use of mouthwash that morning, time of last dose of inhaled medication (asthma and COPD patients only), time of last caffeinated drink, time of last food intake, and time of last vigorous exercise

#### Disease Questionnaires

These will be completed only by patients with asthma and COPD and forms part of their standard clinical assessment in the outpatient setting.

#### Asthma Control Questionnaire

The ACQ is a validated 7-item questionnaire for assessing the level of asthma control over the preceding 7 days. The questionnaire includes five symptom scores, the frequency of rescue bronchodilator use, and a measure of airway calibre (FEV_1_% predicted). Responses are given on a 6-point scale, and the overall score is the mean of the responses (0=totally controlled, 6=severely uncontrolled). Scores over 1.0 are considered indicative of poor control [39,40].

#### Chronic Obstructive Pulmonary Disease Assessment Test

The CAT is a validated 8-item unidimensional measure of health status impairment in COPD. It assists patients and their physicians in quantifying the impact of COPD on the patient’s health. Responses are given on a 5-point scale with a maximum total score of 40. The higher the score, the greater the impact COPD has on the patient’s health; scores of 0 to 9 are considered low impact, 10 to 20 medium, 21 to 30 high, and >30 very high [41].

#### Asthma Quality of Life Questionnaire

The AQLQ is a validated 32-item questionnaire that measures the functional problems (physical, emotional, social, and occupational) that are most troublesome to adults with asthma. Patients are asked to think about how they have been over the previous 2 weeks and respond to each of the 32 questions on a 7-point scale (7=not impaired at all, 1=severely impaired). The overall score is the mean of all 32 responses [42,43].

#### Skin Prick Testing

Asthma participants will have an SPT performed to determine their atopic status. If the participant has had an SPT performed and recorded within the last 3 years, then this result will be used. An SPT is a simple and safe method of testing a person to determine whether or not they have an IgE-mediated allergic response to common inhaled allergens [44]. SPT’s will be performed by trained and experienced respiratory HCPs, who are also trained in resuscitation techniques. Five common aero-allergens will be tested for: grass, house dust mite, aspergillus, cat dander, and dog dander. Atopic status will be demonstrated by a positive SPT (wheal diameter ≥3 mm larger than the negative control).

#### Respiratory Tests

The participants will then continue with the standard respiratory assessment element of their clinic visit, with the addition of the EBC H_2_O_2_ levels measurement performed by the Inflammacheck device, in the order described below. For every test, the following will be recorded:

- the time the test starts and finishes
- the room temperature at the time of the test
- the time of the participants last inhaled medication dose
- the time of the participants last cigarette (if applicable)
- the time of the participants last food and drink intake
- the time of the participants last vigorous exercise
- whether the test was not possible to complete, and the reason for this
- if test was performed, the number of attempts required to complete the test successfully
- any adverse events related to the test that are reported by the patient or noted by clinical staff during the test procedures

#### Exhaled Breath Condensate Hydrogen Peroxide

EBC H_2_O_2_ will be measured using the Inflammacheck device (Exhalation Technology Ltd., Dereham, Norfolk) as specified by the manufacturer’s instructions. For the participant, this involves simple relaxed tidal breathing for 20 breaths into a disposable mouthpiece. Once they have completed this, the research nurse will remove the collection cartridge, which should contain at least 60 µL of EBC. The nurse will then pipette 30 µL onto the Inflammacheck sensor cartridge. The sensor cartridge will be placed back into the device and will give a reading of EBC H_2_O_2_ to the researcher via an attached laptop. The remaining 30 µL will then be pipetted onto a separate reference sensor. This reference sensor will give a separate reading of EBC H_2_O_2_, and will be used to confirm the consistency and reliability of the Inflammacheck sensor. The results of this test will not be disclosed to the patient to avoid bias in effort for subsequent tests. The result will also not be recorded in the clinical notes that are passed to the consulting doctor as they are not intended to inform patient management decisions in this study.

#### Fractional Exhaled Nitric Oxide

FeNO will be measured using a NIOX MINO device (Aerocrine AB, Solna, Sweden,) or equivalent device for measuring exhaled nitric oxide level, as specified by the manufacturer’s instructions and outlined in the American Thoracic Society (ATS) and European Respiratory Society (ERS) standards. This includes collection by controlled exhalation at the recommended expiratory flow rate of 50 mL/s for greater than 6 seconds [45].

#### Spirometry

Spirometry will be conducted using a spirometer conforming to ATS and ERS standards as specified by the manufacturer’s instructions. Participants will inhale rapidly and completely from functional residual capacity, then exhale in an initial blast of exhalation, and then continue exhalation until the end of the test. FEV_1_ (L), FVC (L), and FEV_1_/ FVC ratio will be recorded. FEV_1_ and FVC will be documented as both absolute values and as a percentage of the predicted value [46].

#### Self-Completed Questionnaires

A brief, self-completed questionnaire will be used to evaluate participant’s opinions of the Inflammacheck device on a Likert-type scale. Participants will be asked about ease of use, comfort during testing, perception, and satisfaction of Inflammacheck. They will also be asked to compare Inflammacheck with spirometry and FeNO, stating their preference and which they found easiest to use. At the end of the study, a questionnaire will be used to evaluate HCPs opinions of the different study assessments. Informed consent will be obtained from each HCP to participate within the study. Only HCPs who performed the respiratory tests during the trial on a minimum of 5 patients will be asked to participate. The questionnaire will include a rating for each of the study assessments (spirometry, FeNO, and EBC H_2_O_2_ as measured by Inflammacheck) on a Likert-type scale. HCPs will be asked about “ease of use” of each device, their perceptions of patient experience, and an open-ended question for any further suggestions.

#### Discontinuation of Participants From Study and End of Study

Participants who are unable to perform EBC H_2_O_2_ measurement with Inflammacheck, spirometry, or FeNO will not be withdrawn. The reason for failing to perform these tests will be documented in the case report form (CRF). The end of study is the date of the last participant completing their study procedures.

### Safety Assessment

#### Adverse Event (AE) Definition

An adverse event is any untoward medical occurrence in a participant taking part in a clinical trial that does not necessarily have to have a causal relationship with the device under investigation. An AE can therefore be any unfavorable or unintended sign, symptom, or disease temporarily associated with the use of the device, whether or not this has a causal relationship with the device under investigation.

#### Recording and Reporting of AEs

There are not expected to be any AEs associated with the use of the Inflammacheck device. Only AEs that have a reasonable possibility of being attributable to the device and any other AE considered to be of clinical significance by the principal investigator (PI) as causing harm to the patient will be recorded in the CRF and reported to the sponsor as per their guidelines. We will record all AEs that are observed during all respiratory test procedures as a study outcome. Any AEs that do occur and are considered by the PI to be related to the device will be expedited to the sponsor, research ethics committee (REC), and the device manufacturer within 7 days. Lists of the AEs will be provided to the sponsor when requested.

### Data Handling and Analysis

#### Data Collection and Management

Enrollment into the study will be documented in each participants’ medical notes.

Data collection forms will comprise the following:

- Main CRF, including participant characteristics, SPT results, and respiratory test results.
- Disease control questionnaire (ACQ-7)
- QoL questionnaires (AQLQ and CAT)
- Self-completed questionnaire for participants
- Self-completed questionnaire for HCPs

A bespoke database with preset validation criteria will be created for the study. Data will be entered and checked against the original CRF. Further verification will be done according to frequency and pattern of errors. All data verification will be carried out by the research team and then by the sponsor during their monitoring procedures.

#### Data Analysis

All participants with an EBC H_2_O_2_ result as measured by Inflammacheck will be analyzed. Subgroup analyses may be carried out if there are sufficient numbers of patients with particular characteristics, for example, smokers versus nonsmokers. Demographics or baseline characteristics of each of the study groups (COPD, asthma, and control) will be produced, as well as summaries for all groups combined. Normally distributed continuous variables will be summarized by the mean and standard deviation, whereas the median and interquartile range will be preferred for non-normally distributed continuous variables. The number and percentage of subjects in each category will be recorded for categorical variables.

#### Primary Analysis

To establish our primary objective, the primary analysis will be a comparison of EBC H_2_O_2_ values, as measured by the Inflammacheck device between each pair of the three main study groups (COPD, asthma, and control). It is expected that the EBC H_2_O_2_ will have a positively skewed distribution. To allow for the skewed distribution, one approach would be to analyze the data on a log transformed scale and to compare between groups using analysis of variance (ANOVA), with post-hoc tests performed to compare between pairs of groups. It is possible that there may be EBC H_2_O_2_ measurements below the lower detection limit, and thus, the previous approach may not be practical. If there are measurements below the detection limit, this will be dealt with using nonparametric methods. The Kruskal-Wallis test will be used to compare between the three groups, with the Mann Whitney test used to compare between pairs of groups. When comparing between pairs of groups, a Bonferroni correction will be applied to allow for multiple testing.

#### Secondary Analyses

There will be a comparison of EBC H_2_O_2_ values, as measured by the Inflammacheck sensor and the values measured by the reference sensor. This will determine whether EBC H_2_O_2_ is being measured consistently and reliably by the Inflammacheck sensor, giving confidence for its use in future versions of the Inflammacheck device. The association between the EBC H_2_O_2_ measurements and a number of other parameters will also be examined. Associations will be examined with the following:

- FeNO (both high and low levels)
- Disease severity
- Disease control measures
- Disease QoL measures
- Spirometry measures (FEV_1_, FVC)
- Atopic status (in asthma participants only)
- Items identified in the pretest checklist

Associations with continuous variables will be examined using either Pearson or Spearman rank correlation (as appropriate). Associations with categorical measures will be assessed using ANOVA (using transformed H_2_O_2_ values) or the Kruskal-Wallis test.

The percentage of attempted tests that *failed* for each test will be quantified. The association between patient characteristics and this outcome will be examined. Assuming patient characteristics are categorical in nature, the chi-square test or Fisher exact test will be used to examine associations with this outcome. The secondary analyses will be performed for all subjects combined and also for each study group separately.

#### Procedure for Dealing With Missing and Spurious Data

The analysis will include only measured data values, with missing values omitted from the analysis. No imputation of missing data will be performed. The data will be examined for outlying values. Where possible, these will be retained in the data analysis and their influence minimized by a data transformation or a nonparametric approach. If such approaches are not practical, the analysis of the primary outcome will be performed twice, with and without the outlying values.

### Ethics

#### Participant Confidentiality

The study staff will ensure that the participants’ anonymity is maintained. The participants will be identified only by initials and a participant’s ID number on CRF and any electronic database. All documents will be stored securely and only accessible by study staff and authorized personnel. The study will comply with the Data Protection Act that requires data to be anonymized as soon as it is practicable to do so.

#### Other Ethical Considerations

The study will not be initiated before the protocol and all study relevant material such as the informed consent forms and PISs have received approval or favorable opinion from the REC and the respective National Health Service (NHS) research and development (R&D) departments. Any changes to protocol or relevant study documents will be approved by the sponsor. Should an amendment be made that requires REC approval, as defined by REC as a substantial amendment, the changes will not be instituted until the amendment has been reviewed and received approval or favorable opinion from the REC and R&D departments. A protocol amendment intended to eliminate an apparent immediate hazard to participants may be implemented immediately providing that the REC are notified as soon as possible and an approval is requested. Minor amendments as defined by REC as nonsubstantial amendment, may be implemented immediately; and the REC will be informed. All participants will have adequate time to consider participation in the study, as per Good Clinical Practice (GCP) guidelines.

Patients who are already enrolled in other research trials will be invited and allowed to participate in the study if they so wish. This was discussed with our patient and public involvement (PPI) representatives who felt that these patients should also have the opportunity to participate in the study and should not be excluded. There is a possibility that the study procedures reveal potential new, previously unknown disease pathology. This would be more likely to occur in our healthy controls. If such a circumstance occurs, then the participant will be told of the results and immediately referred to the most appropriate NHS department for further review. With the participant's consent, a letter will be written to their general practitioner explaining the findings.

#### Informed Consent

It is the responsibility of the investigator, or a person designated by the investigator (if acceptable by local regulations), to obtain written informed consent from each person participating in this study after adequate explanation of the aims, methods, anticipated benefits, and potential hazards of the study using the PIS. The consent process will be documented in the participant’s notes.

The process for obtaining participant informed consent will be in accordance with the REC guidance and GCP and any other regulatory requirements that might be introduced. The PI or delegate and the participant or any other legally authorized representative shall both sign and date the informed consent form before the person can participate in the study. The participant will keep the PIS and a copy of the signed and dated consent form. The original will be retained in the trial master file. A second copy will be filed in the participant’s medical notes and a signed and dated note made in the notes of when the PIS was provided and that informed consent was obtained for the study.

The decision regarding participation in the study is entirely voluntary. The investigator or their nominee shall emphasize to them that consent regarding study participation may be withdrawn at any time without penalty, or affecting the quality or quantity of their future medical care, or loss of benefits to which the participant is otherwise entitled.

### Patient and Public Involvement

PPI involvement in this study has been sought from patients with first-hand experience of living with chronic respiratory disease. Through face-to-face meetings, email, and telephone contact, we have discussed the concept, impact, and details of the study with our asthma patient representatives from the Wessex Asthma Network and our COPD patient representatives from local British Lung Foundation groups. These people have lived with severe asthma and COPD and been involved in previous research studies. They contributed to developing the key questions and setting our study objectives, ensuring that we answer the questions that are relevant to people suffering from airways disease. They have also helped us with patient recruitment design and the implementation of the Inflammacheck test within a standard clinical visit so as to minimize delays for patients who agree to participate in the study. They have helped design the questionnaire that will be used to record the participant experience of the device, PIS and have coauthored the lay summary.

## Results

Recruitment to the EXHALE pilot study is ongoing. It is anticipated that results will be available by early 2018.

## Discussion

The EXHALE pilot study will provide an evaluation of a new method of measuring EBC H_2_O_2_. It will assess the device’s (Inflammacheck) consistency and accuracy of measurement of EBC H_2_O_2_ and its ability to distinguish airway inflammation in asthma and COPD compared with healthy controls. The data collected will allow us to develop the device further in response to participant feedback and prepare for future longitudinal studies that will assess its capability for detecting asthma and COPD exacerbations.

## Abbreviations

ACQ: Asthma Control Questionnaire
AE: adverse event
ANOVA: analysis of variance
ATS: American Thoracic Society
AQLQ: Asthma Quality of Life Questionnaire
CAT: COPD Assessment Test
COPD: Chronic Obstructive Pulmonary Disease
CRF: case report form
EBC: exhaled breath condensate
ERS: European Respiratory Society
FeNO: fractional exhaled nitric oxide
FEV_1_: forced expiratory volume in 1 second
FVC: forced vital capacity
GCP: Good Clinical Practice
GINA: Global Initiative for Asthma
GOLD: Global Initiative for Chronic Obstructive Lung Disease
H_2_O_2_: hydrogen peroxide
HCP: health care professional
NHS: National Health Service
PI: principal investigator
PIS: participant information sheet
PPI: patient and public involvement
QoL: quality of life
R&D: research & development
REC: research ethics committee
ROS: reactive oxygen species
SPT: skin prick testing
